# Infrastructure, ontology and meaning: The endogenous development of economic ideas

**DOI:** 10.1177/03063127211011524

**Published:** 2021-04-22

**Authors:** David Pinzur

**Affiliations:** 1 London School of Economics and Political Science, London, UK

**Keywords:** markets, infrastructure, derivatives, market ontology, sociology of knowledge

## Abstract

In contrast to work showing exogenous social influences on the production of economic ideas, this article asks how a market’s own infrastructure can endogenously shape practitioners’ economic perspectives. It investigates this question by comparing the evolution of opposed views on speculation across two 19th-century American futures markets. The analysis locates the origins of this divergence in features of the grading, receipting and contracting processes that linked these new derivative markets to underlying agricultural markets. This connective infrastructure both made possible new speculative practices and established market ontologies from which traders theorized the economic significance of those practices. These ontologies served as distinct cores around which incompatible constellations of ideas – including beliefs about price relations between spot and futures markets, the character of the global market and the motives and capabilities of speculators – were elaborated.

## New markets, new questions

The decades following the Civil War in the United States saw the rapid growth of a new economic institution: futures markets. These markets were derived from, and operated in conjunction with, extant agricultural markets. The commodities bought and sold on futures markets were standardized contracts that promised the exchange of agricultural goods at pre-determined qualities and prices on some future date. While originally an adjunct to traditional markets, derivatives quickly grew far larger than the underlying cash, or ‘spot’, trade (Markham, 1987). This growth was due to the possibility futures offered for purely speculative trading. Since speculative trades almost never required the exchange of real goods, they could reach extreme levels: for example, on a single day in 1890, speculators on the New York Produce Exchange traded futures contracts representing two and a half times the total amount of wheat that would reach their spot market that entire *year* (US House of Representatives, 1892: 13).

This explosion of speculation prompted questions from both practitioners and observers. Was such a large amount of speculation safe? Did speculative trades – including ‘short sales’ where traders contracted to sell goods they did not yet own – raise spot prices, lower them or not affect them at all? What exactly made speculation that ended without the exchange of goods any different from illegal gambling? These questions were not only economic, but political, legal and cultural. Novel speculative practices were scrutinized by the press, state and federal legislatures and courts. In fact, a number of extant sociological analyses of 19th-century futures markets, focused particularly on the Chicago Board of Trade, have argued that this scrutiny influenced traders’ and exchanges’ answers to the above questions: Exchanges painted futures speculation as necessary, salutary and subsidiary to agricultural markets with the express purpose of appeasing powerful audiences and quelling the threat of prohibition or restrictions (Cronon, 1992; De Goede, 2005; Fabian, 1999; Lurie, 1979; Stäheli, 2013).

The current article challenges two aspects of this explanation. First is the implicit claim that all American exchanges in this period made the same defense of speculation. A close examination of economic discourses on two leading exchanges – the Chicago Board of Trade (CBOT) and New Orleans Cotton Exchange (NOCE) – reveals stark disagreement on several key issues, including the relation between prices on spot and futures markets, the character of the global market and the motives and capabilities of speculators. Second is the argument that exogenous social factors can fully explain this observed difference in theoretical discourse. In fact, Chicago and New Orleans produced opposite perspectives despite facing remarkably similar pressures and threats in the development of their new markets.

I argue instead that practitioners’ economic theories emerged endogenously from the foundational infrastructures – integrated ensembles of technological devices, standards, classifications, protocols and material arrangements (Pardo-Guerra, 2015: 6) – that connected futures markets and underlying spot markets on each exchange. My findings show that differences in these infrastructures produced distinct market ontologies (Jensen and Morita, 2017) – beliefs regarding the objects that existed in the market and their properties – from which practitioners came to understand speculation. These ontologies, while not forcing practitioners to adopt any particular economic ideas, acted as ‘limiting causes’ (Wright et al., 1992) – conceptual cores that enabled or precluded the elaboration of certain, broad perspectives on the market (Biernacki, 1997). The article thereby outlines a previously unrecognized, endogenous process by which market infrastructures not simply enable new market practices, but also shape how those practices are understood. This argument has implications for the study of infrastructure, sociology of markets and sociology of knowledge.

## Spot and futures markets

As a necessary prelude to this analysis, I begin by sketching the central features of spot and futures markets in the 19th century. Traders in spot markets bought and sold agricultural commodities in traditional fashion, exchanging cash and goods ‘on the spot’. Traders in futures markets, on the other hand, bought and sold contracts, which promised the exchange of agricultural goods at specified quantities, qualities and prices on some future date. The key to making these contracts tradeable was establishing widespread standardized grades (e.g. #1 spring wheat, middling cotton) and measures (i.e. a bushel or bale), which could be inscribed on contracts. For example, a contract made in February might specify the sale of 500 bushels of #1 spring wheat at a price of $1 per bushel on any day in June. These standardized elements linked physical commodities on the spot market with contracts on futures market and also made contracts fungible and liquid commodities in their own right.

With these elements in place, a trader who held a futures contracts could settle the obligation in two ways: by *delivery* or by *setting off*. In the former, a trader would settle the contract by delivering or receiving at the agreed-upon price the contractually-promised physical commodity or a warehouse receipt representing its ownership. In setting off, speculators would exit their obligation not by completing the promised deal, but by trading their stake in the contract to another speculator who would assume the contractual duty to buy or sell. This can equivalently be viewed as a trader canceling one contractual obligation (e.g. to buy) by assuming its opposite (i.e. to sell) in a second trade, making their own involvement redundant. In short, settlement by setting off relieved a contract holder of the responsibility to deliver or receive actual goods. This had great utility for speculators, who, so long as they had ‘set off’ any obligations by the time a contract came due, never had to handle any physical commodities. This made speculation cheap. Speculators avoided the costs associated with buying, storing and transporting goods and only had to put up a small margin to enter into a contract. For these reasons, setting off quickly became the dominant method for settling contracts and the core mechanism behind an expansive speculative trade. (Though, as we will see, while settlement by delivery was rare, it was not unimportant.)

The economic impact of the speculative boom unleashed by setting off was debated fiercely in the decades following the Civil War. The next section briefly reviews – and finds wanting – extant arguments that 19th-century exchanges’ perspectives on the topic were shaped solely by the exogenous social pressures of the time.

## The CBOT and the insufficiency of exogenous factors

Extant research on the development of 19th-century economic theory has taken a sociology of knowledge-style approach that traces the impact of social context on practitioners’ ideas.^1^ Much of this work has been focused on one of the two cases presented here, demonstrating how the Chicago Board of Trade’s economic discourse emerged in response to challenges over the legitimacy of setting off. These challenges stemmed from the fact, noted above, that setting off allowed traders to settle contracts without delivering any goods. Opponents argued that this made setting off less a legitimate mercantile practice than a cover for gambling on the price of agricultural goods. The damage of the association with gambling had multiple facets. Culturally, speculation was stigmatized as a morally corrosive danger to citizens ( De Goede, 2005; Fabian, 1999). Legally, the gambling issue raised questions regarding the validity and enforceability of speculative contracts (Levy, 2006; Lurie, 1979). Politically, the language of gambling fed agrarian and populist suspicion of exchanges and provoked legislative threats, including some 200 bills designed to regulate or prohibit futures and options trading introduced into Congress between 1880 and 1920 (Cronon, 1992; Markham, 1987).

Scholars have argued that the CBOT crafted their economic discourse about futures speculation to relieve the pressure they faced in this contentious environment (Banner, 2016; De Goede, 2005; Fabian, 1999; Levy, 2006; Stäheli, 2013). To address the cultural problems, exchanges defined speculation as a natural practice, developed by market experts, for dealing with the unavoidable risk of price fluctuations in the free market (Fabian, 1999; Stäheli, 2013). Whereas gamblers chased a thrill in artificial sources of risk such as the roll of the dice or turn of the card, speculators ‘only worked with risks in order to reduce them’ (Stäheli, 2013: 53). This moral distinction helped to quell some political anger, as did the argument that speculation helped both farmers and consumers by eliminating disruptive, seasonal price cycles (De Goede, 2005; Fabian, 1999). Finally, exchanges hung their legal defense on the claim that all speculative contracts were fully binding. Delivery, though rare in practice, could always be demanded by any contract holder, which distinguished contracts from mere wagers (Levy, 2006; Lurie, 1979). These economic ideas emerged as part of the Board’s effort to maintain legitimacy amid numerous exogenous challenges.

However, comparison with the New Orleans Cotton Exchange suggests that this story, while valuable, is incomplete. First, the CBOT-focused, single-case analysis fails to recognize the diversity of ideas about speculation that existed among 19th-century exchanges. While leaders of the CBOT and NOCE both subscribed to the general defense of speculation above, they persistently disagreed on important particularities, including the relation between spot and futures markets, their price influence on each other, the cohesiveness of the global market and the character of speculators. Further, these differences throw into question the adequacy of the sociology of knowledge-style explanation on offer. Practitioners on the CBOT and NOCE produced opposed theoretical viewpoints despite their similar economic, cultural and political environments. Both exchanges were early adopters of futures trading forced to publicly defend the legitimacy of their markets (Bouilly, 1975; Ellis, 1973; Markham, 1987). Both were located within agricultural regions where powerful branches of the Farmers Alliance fought legislative and cultural battles against them (Hicks, 1931). The state legislatures of Illinois and Louisiana both sought to prohibit speculative short selling, the former in 1874, the latter in 1888. At the federal level, the vast majority of bills aimed at futures trading – including 1892’s Hatch and Washburn Bills, the most serious dangers – threatened both exchanges equally.^2^ Finally, while the CBOT was involved in more court disputes than the NOCE, the details of this litigation suggest that any impact on the development of economic ideas would have been mixed.^3^ As a whole, these factors indicate that the theoretical divergence seen in Chicago and New Orleans was not based solely in social milieu, but may also have reflected the impact of factors *endogenous* to the market.

## Theoretical divergence and endogenous difference

Making sense of this endogenous influence requires theoretical perspectives that view markets as heterogeneous constructions, composed of intertwined social relations, material devices, technical processes *and* economic ideas (Çalışkan and Callon, 2010; Callon, 2007). Such approaches allow us to understand economic theories as one of many linked, sociomaterial components that format markets and enable practice under conditions of pervasive uncertainty (Beckert, 1996, 2016).

One lesson learned from positioning economic ideas in this light is that practitioners’ viewpoints reflect their everyday, device-mediated interactions with the market (Aspers, 2007; Knorr Cetina and Preda, 2007). Visual technologies such as screens, charts and figures act as ‘scopes’ that ‘project market reality’ (Knorr Cetina, 2003: 7) and provide actors with ‘new ways to think about … interrelationships between market components’ (Pryke, 2010: 452). Changes in market devices can lead to radically new economic ideas. Prices conveyed by voice, on ticker tape or on computer screens each provoke different conceptions of the market: as a collection of individual players versus a singular unit, something to be ‘found’ versus something omnipresent or a definite location versus a global flow (Knorr Cetina, 2003; Preda, 2006; Zaloom, 2004). Preda (2006, 2009) demonstrates how the interactions enabled by a new market device (i.e. the stock ticker) formed the grounds for a new, fully-fledged perspective on financial markets (i.e. Chartism), complete with novel analytical categories (i.e. the ‘new tops’, ‘head and shoulders’ or ‘double bottoms’ analysts discovered in their charts).

A second insight from viewing markets as heterogeneous constructions is that economic ideas are not detached representations of markets, but tools for making active interventions within them. This perspective is developed in two related areas of the literature. First, work on the performativity of economics has demonstrated that economic theories shape material features of markets (Callon, 1998), from the design of calculative aids (MacKenzie, 2006), to the physical layout of markets (Garcia-Parpet, 2007), and even the bodily sensations of traders (Lee and Martin, 2016; Wosnitzer, 2016). This process of materialisation instantiates economic theory ‘beyond human minds’ (Callon, 2007: 323), occasionally pushing market actors’ behaviours to conform more closely to theory’s predictions (MacKenzie, 2006). Second, research has uncovered how traders use economic models primarily as practical tools, or ‘unfolding elements of situational practices’ (Svetlova, 2018: 57), rather than objectively true pictures of economic reality. Models are constructed via *bricolage* (MacKenzie, 2003), cobbled together from multiple sources to help market actors achieve particular, profit-oriented goals, given a unique set of (often organisational) resources and (often regulatory) constraints (MacKenzie and Spears, 2014; Sismondo, 1999). Traders using models as tools tend to care little about theoretical cohesiveness, combining distinct and sometimes contradictory, techniques and analyses based on the needs of the situation at hand (Leins, 2018; MacKenzie, 2003). Their desire is not to understand the market – a goal that many practitioners believe is unattainable – but rather to allow ‘practical judgment’ despite pervasive ignorance (Smith, 2011; Svetlova and Dirksen, 2014).

Each of these perspectives describes a different way that sociomaterial practices endogenous to the market interact with ideas about those markets. The first proposes a process in which ideas are a phenomenological outgrowth of interactions within market settings; the second, one where ideas are pragmatically applied in the service of achieving market-oriented goals. In both, practices and ideas are linked through everyday interactions: models and representative devices are referenced day-to-day, if not minute-to-minute, in fast-moving decision-making environments; performativity and bricolage often involve everyday elements, including ready-to-hand calculative and representative devices as well as organisational arrangements. This focus on the level of market practitioners’ everyday interactions, however, has meant that the sociomaterial ensembles that underlie and support those interactions – market infrastructures – have remained understudied. How might these integrated sets of categories, standards, devices, mechanisms and protocols (Pardo-Guerra, 2015) themselves endogenously shape the production of economic ideas?

Existing scholarship on infrastructure offers, if not clues, then at least encouragement that pursuing this question might produce answers. This is because infrastructural analyses echo many of the themes found in the above literature. Like representative and calculative devices, infrastructures harmonize actions across diverse communities of practice, enabling the emergence of new, higher-order actions (Barry, 2006; Bowker, 1994; Bowker and Star, 2000; Edwards, 2003; Star and Ruhleder, 1996). They act as ‘world-ordering arrangements’ (Carse, 2012: 543) with significant material and epistemological consequences for market actors (Muniesa, 2007; Pinzur, 2016). Infrastructures also serve as tools for solving fundamental market challenges, enabling acts of coordination within an environment of uncertainty (Beckert, 2009, 2016; Pinzur, forthcoming). Like economic models, infrastructures are designed to solve problems facing market action, for example, recruiting actors into a new market (Guseva and Rona-Tas, 2014), enabling cross-border payments (Jeffs, 2008) or guaranteeing the consummation of deals (Millo et al., 2005). Infrastructures, though operating in the market’s background, can have similar functions and consequences as everyday calculative and representative devices.

But despite these general confluences, the particular endogenous connection between infrastructures and economic ideas remains unspecified. How can sociomaterial elements which are *not* ready-to-hand, focal points of everyday market behaviour, but which rather transparently underlie and enable these interactions, shape the development of economic ideas? This is the core theoretical question I address here. My answer, developed in the findings and discussion below, is that infrastructures produce market ontologies (Jensen and Morita, 2017) which act as ‘limiting causes’ (Wright et al., 1992), non-deterministically guiding practitioners’ economic perspectives into cohesive constellations (Biernacki, 1997).

Data come from CBOT and NOCE annual reports, publications and committee reports, as well as exchange leaders’ testimonies and memorials before the 52nd and 61st Congresses opposing bills that threatened speculation in futures. These data show exchange leaders explaining and justifying futures speculation in economic terms, referencing the value of the futures market, its benefits and dangers, and the likely impact of proposed regulations or prohibitions. Of course, public arguments were made in the context of debates over the exchanges’ legitimacy and economic future, and must be read within the political frame of this contestation. Still, the general continuity of messaging across exchange members’ public statements and private communications suggest that their discourse was not purely a cynical response to external pressure. We can be confident that these statements, in large part, represent exchange members’ genuine efforts to understand the nature of the brand-new market they faced.

## Findings

My findings are presented in three sections, each of which draws a different contrast between the two cases. These illustrate (1) how differences in the exchanges’ infrastructures for the delivery of physical commodities established distinct market ontologies, which (2) served as bases for practitioners’ divergent understandings of settlement by setting off and (3) placed limiting conditions on their developing views of speculation more generally, contributing to the exchanges’ contrasting perspectives on topics such as price relations between spot and futures markets, the character of the global market and the motives and capabilities of speculators. In this way, the analysis shows how exchanges’ ideas about the novel, controversial practices of mass speculation were endogenously shaped by the pedestrian infrastructures that linked spot and futures markets.

### Delivery infrastructures and market ontologies

I begin by illustrating differences in the infrastructures that the CBOT and NOCE used to settle contracts by delivery, including in their grading, receipting and contracting components. I then argue that these infrastructural differences produced distinct market ontologies on the two exchanges: On the CBOT, graded bushels were considered an extant reality, predictably linked to contracts via warehouse receipts, while on the NOCE graded bales remained only a potentiality, making the relation between commodities and receipts unpredictable (see Table 1).

**Table 1.** Infrastructures for settlement by delivery.

Component	CBOT	NOCE
Grading	Grading upon storage	Grading upon exchange
Receipting	References graded goods	References *sui generis* goods
Contracting	Single-grade contracts	Multiple-grade contracts
Pricing	Established in contract	Established by quotation committee

Commodity grading was the first component of the delivery infrastructure produced on both exchanges. In Chicago, it was introduced by owners of grain warehouses, who used grades as a way of maximising storage capacity and profit. Commodities would be graded upon entering into store, then combined in large bins with shipments of like quality from other sources. Warehouses would issue receipts that entitled the holder to remove an equal quantity of the same grade of grain. In 1870, the state of Illinois assumed responsibility for grading and receipting, which both indemnified sellers against legal disputes and increased trust in the accuracy of the grades worldwide (Cronon, 1992; Hill, 1990; Lee, 1938; Lurie, 1979). Contracts could be made for any single grade of grain, whose price was specified unambiguously in the contract. Settlement by delivery could then be made by transferring either physical bushels of the properly-graded wheat or state-certified warehouse receipts which represented ownership of the same held in storage. The latter option was far more common and essentially equivalent to the former. Receipts represented ‘property that can be realized upon without delay’ (New York Cotton Exchange et al., 1892: 45); delivering them transferred legal ownership, while avoiding the hassle of unnecessarily transporting physical goods (US House of Representatives, 1892).

Taken as a whole, this delivery infrastructure imbued grain, receipts and contracts with a unique set of characteristics. First, crops entering warehouses would undergo an ‘ontological mutation’ (Callon, 2007: 337), transforming from *sui generis* goods to instances of a more general class of object. Receipts then could signify ownership of these new categorical objects, while single-grade contracts could index and promise them. The ‘graded bushel’ became an extant, physical object that could be pointed to, promised and represented within the CBOT’s delivery infrastructure. Thanks to these features, settlement by delivery could be accomplished through the simple transfer of receipts and money, without the grain itself being involved.

The NOCE’s infrastructure also included standardized and well-established grades, but differed in that grading, and the ‘ontological mutation’ it entailed, occurred not upon cotton’s entry into store, but only upon delivery, *after* a sale was made.^4^ At that point, both buyer and seller would hire expert inspectors to sample the delivered bales and negotiate their grades. This difference in the timing of grading had consequences for other components of the delivery infrastructure. For example, receipts represented ownership not of a general class of graded commodity, but rather of specific, ungraded bales of cotton. Further, the impossibility of knowing the grade of any given bale prior to delivery necessitated complementary contracting and delivery procedures. All contracts were *written* for a single grade of cotton (‘middling’), yet could be *fulfilled* through delivery of a range of grades rather than any single one – nine different grades of cotton could legally be delivered in satisfaction of a futures contract (Boyle, 1934; Garside, 1935; Sherman, 1934). This arrangement required an additional ex-post mechanism to adjust the price paid for delivered cotton judged to be better or worse than ‘middling’ quality. An Exchange body called the Committee on Future Quotations made these adjustments, meeting at the close of each trading day to determine, based on sales and tone in the day’s spot market, the appropriate premium above or discount below contract price to be applied for each grade.^5^

The delivery infrastructure on the NOCE thus produced a market ontology distinct from that found on the CBOT. Physical bales remained *sui generis* commodities throughout the futures trading process, with their transformation via grading being realized only at the point of delivery. Warehouse receipts could only signify ownership of ungraded bales, not the graded versions referenced in futures contracts. For NOCE futures traders, the ‘graded bale’ was a meaningful concept, but not a physical reality. Contracts promised a range of potential, not-yet-extant, physical goods. As a result of these infrastructural features, settlement by delivery could not occur simply through the exchange of receipts, but required the delivery of actual cotton, followed by the negotiation of a grade and determination of a final price.

It is critical to reiterate here that these differences in delivery infrastructure were largely inconsequential for the practical operation of futures markets. Settlement by delivery was exceedingly rare. Though neither exchange kept records on the topic, contemporary reports suggest that only a tiny percentage of trades were settled by delivery (Cronon, 1992; Fabian, 1999; Levy, 2006; Stäheli, 2013). Further, the alternative process of settlement by setting off was unaffected by these differences in delivery infrastructure. On both exchanges, traders who had bought a given quantity of futures contracts could set off their trade simply by turning around and selling an equal quantity of the same contract, or vice versa.^6^ Once this had happened, the trader would no longer have any outstanding contractual obligations and would simply settle his profit or loss following from the difference in purchase and sale prices. Delivery infrastructures were not practically involved.

Given this, one might assume that these infrastructural differences would have no impact on the production of economic ideas about the process of setting off. The following section shows otherwise. Though speculative traders did not engage with the mechanisms of the delivery infrastructure, the ontologies that those infrastructures established served as discrete bases from which traders produced divergent understandings of setting off. On the CBOT, setting off was conceptualized as a detour of physical goods through the futures market via ‘theoretical exchange’, while on the NOCE it was a form of ‘insurance’ occurring in parallel to spot market exchanges.

### Setting off: Theoretical exchange or insurance?

As shown above, the ontology produced through the CBOT’s delivery infrastructure featured unambiguous, one-to-one linkages between graded spot commodities, warehouse receipts and single-grade futures contracts. In this system, a trader who had bought and sold futures for equal quantities of the same grade of wheat could, if they desired, receive graded bushels or receipts in fulfillment of the first contract, then immediately hand these over to settle the second. All of the elements involved had fixed characteristics across markets, enabling settlement by delivery of goods or receipts to occur predictably.

Based on this predictability, the Board argued that setting off was merely a simplified version of what would otherwise occur through a long string of real deliveries. Presenting the case before Congress in 1892, CBOT director Howard Aldrich made this argument with respect to a hypothetical ‘Mr. A’ and ‘Mr. B’, who had entered into two opposing contracts with each other. In such an instance:

There are two contracts exactly alike, the only difference between these two contracts is the difference in price, and the fact that one party is a buyer in one and a seller in the other. … Now the result is exactly the same whether the property is delivered … or one contract offset against the other, and so A and B settles [sic] … it is a very great advantage in the markets that contracts could be adjusted that way. (US House of Representatives, 1892: 74)

Delivery in this case *could* have been made, but was unnecessary: Since prices and goods were established entirely by contract and bushels had been assigned unalterable grades, delivery would be rote. Setting off simply removed the predictable – and redundant – transfer of goods or receipts, while retaining the predictable – but necessary – exchange of money.

**Figure 1.**
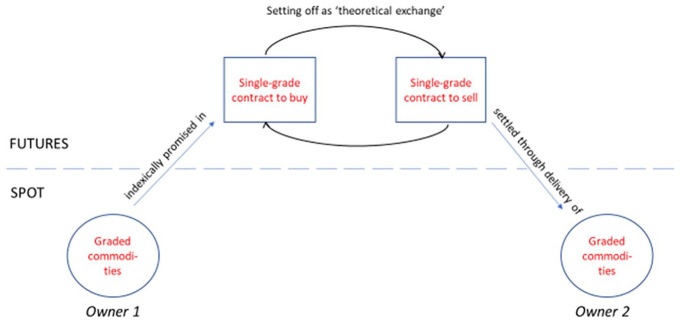
Setting off as ‘theoretical exchange’ on CBOT.

The same reasoning held in more complicated cases, where contracts were offset among multiple parties. Speaking in 1908, Board President Hiram Sager described this in particularly telling language, noting that while physical goods in this process did not ‘actually change hands’, they did ‘*theoretically* do so’ (Sager, 1908: 8, emphasis original). Real commodities could be promised in a futures contract and ‘theoretically’ traded multiple times among speculators before ending up back in the spot market at the end of the chain. Language in a Board-distributed pamphlet crystallized this perspective, describing one party to an offset – a speculative trade which, recall, involves only the exchange of contracts, not commodities – as having ‘bought back’ the wheat they had sold previously (Smith, 1919: 7). Rings of offsets offered a way for ‘actual property’ to be ‘sold and resold a thousand times’, but the chain inevitably ended with ‘the property finally pass[ing] into consumption’ (New York Cotton Exchange, et al., 1892: 46) (see Figure 1).

From the CBOT’s perspective, setting off thus maintained a fundamental, if unseen, connection to agricultural goods. All transactions, including those settled through setting off, were ‘based upon property – tangible, defined, accessible and convertible’ (US House of Representatives, 1892: 157, emphasis altered). Setting off was a conceptual detour within a larger spot market trade – the delivery that was *theoretically* made in setting off was embedded within a delivery that was *actually* made elsewhere. This explanation undoubtedly was motivated in part by the effort to distance exchange practice from illegal gambling (Cronon, 1992; De Goede, 2005; Fabian, 1999; Lurie, 1979). But it was – just as importantly – made possible by the market ontology produced through grading, receipting and contracting processes in the Board’s delivery infrastructure. Since contracts referenced physically present, graded commodities, futures transactions could always refer back to a spot market reality; the ability of receipts to unambiguously connect actual grain to futures contracts enabled market actors to predictably play out chains of hypothetical future trades. Together, these characteristics made thinkable the notion that setting off was equivalent to a ‘theoretical exchange’ of goods.

By contrast, the NOCE’s infrastructure for settlement by delivery precluded any conception of setting off as a simplified version of spot market transactions. Recall that the NOCE did not grade cotton until the point of delivery: ‘graded bales’, at the moment of a speculative trade, remained a mere potentiality, unable to be indexed in a receipt that could be delivered in fulfillment of a contract. Further, contracts, though identical, did not promise a single type of spot market good. In contrast to the CBOT’s unambiguous, one-to-one relations, the connections between infrastructural components on the NOCE were contingent and multiple. The resultant unpredictability in settlement by delivery meant that the CBOT’s perspective on setting off – as a simplified, ‘theoretical’ version of what otherwise would occur via delivery – was absurd on the NOCE, where the outcomes of long chains of delivery could not be predicted ahead of time.

Given the impossibility of unambiguously connecting the spot and futures markets, NOCE members understood setting off as one component of a process that played out across the two markets in parallel: insuring trades in spot commodities through hedging in the futures market (Thompson, 1908).^7^ Insurance through hedging involved a spot trader taking up a complementary, speculative position in the futures market as protection against risk. In this arrangement, any potential loss in the spot market (e.g. from the price of spot cotton dropping while a seller looked for a buyer) would roughly be offset by profit in the futures market (e.g. from having sold cotton short and profited from the price decline). For dealers or manufacturers engaged in a hedge, ‘the contract for the future delivery of cotton … is an insurance policy, protecting from loss by reason of fluctuations in price, just as his fire or marine insurance policy protects him from loss by fire or water’ (Thompson, 1908: 7). This insurance function was, according to Exchange President William Thompson, ‘the greatest value of the future contract … and the largest use to which the future contract is put’.^8^

**Figure 2.**
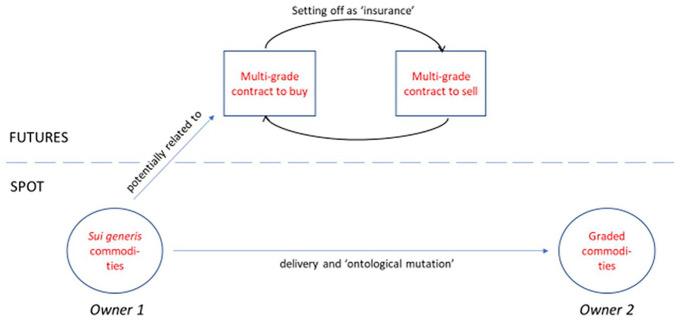
Setting off as ‘insurance’ on NOCE.

In this process, setting off was conceptualized not as a ‘theoretical delivery’ of spot goods, but simply as the means by which a spot trader would close his parallel insurance policy. A spot buyer who had taken a hedge position as insurance, would, upon the sale of his cotton, ‘buy back his future contract, its protective mission having expired’ (Thompson, 1908: 12). Note the subtle, but important shift in language from that seen on the CBOT – the hedger, in setting off, does not buy back his cotton, but his *contract.* Setting off demonstrated the separation, not the connection, between spot and futures markets. As President Thompson noted: ‘All the traders who use the contract as a hedge against such actual [spot market] transactions, have the intention to receive and deliver actual cotton, *but not on the contract they have bought or sold*’.^9^ The ambiguous relation between futures contracts and physical bales thus produced a conceptual – in additional to practical – segregation of setting off from delivery (see Figure 2).

The exchanges, using the dissimilar market ontologies established in their delivery infrastructures, came to divergent understandings of the process of setting off: theoretical exchange and a detour for stable, spot goods on the CBOT versus closing out an insurance policy in a parallel market on the NOCE. In the next section I trace how these perspectives formed the cores of distinct constellations (cf. Biernacki, 1997) of ideas about speculation that diverged in their views of (1) price relations between spot and futures markets, (2) the character of global markets and (3) the motives of speculators. By analysing how these as yet untheorized differences articulate with exchanges’ core positions on setting off, I show how market ontologies can enable or preclude certain directions of theoretical elaboration, serving as ‘limiting causes’ (Wright et al., 1992) in the construction of economic ideas.

### From setting off to speculation: Constellations of economic ideas

The CBOT’s ‘theoretical exchange’ perspective served as the core element in a constellation of compatible economic ideas that included beliefs that prices in futures markets were uni-directionally rooted in spot market dynamics, global markets were integrated and homogenous and speculators were uniformly calculative (see Table 2).

**Table 2.** Perspectives on the characteristics of speculation.

Topic	CBOT	NOCE
Price relations	Uni-directional	Bi-directional
Global market	Integrated	Balkanized
Speculators	Calculative	Affective

We can see this first in its view on price relations between spot and futures markets. Recall, the CBOT maintained that futures contracts promised, and ‘theoretically’ delivered, actually-existing, graded bushels. In line with this belief, it also understood futures prices as reflections of the supply and demand for those same, actual bushels. The Board, for instance, referred Congress to the example of European speculators, who ‘encouraged by the prospect of a very large demand to be caused by a partial failure of their own crops, advanced their bids from day to day’ (New York Cotton Exchange et al., 1892: 51). Active and continuous buying and selling for future delivery forced speculators to assume the role of ‘far-seers, informing themselves of supply and demand throughout the world’^10^ in order to maintain their profits. As a result, ‘except possibly for brief periods, prices of farm products under the operation of this [futures] system are in harmony with the law of supply and demand’ (US House of Representatives, 1892: 157).

The uni-directional reductionism of this view aligns with a belief that speculators are unable to sway market price from the level properly established by supply and demand. The Board, protesting an 1892 anti-futures bill, explained this with reference to a ‘proper price’ at which supply and demand would perfectly be balanced:

The speculator, by his judgment and action, performs the needed service of making actual market price conform to this desired ‘proper price’. He must do so. The condition of his existence as a successful speculator is, that he sees, and conforms his action to, the best interests of producer and consumer. His action cannot influence the ‘proper price’; supply and demand regulate that. His action does determine the actual market price, and only as it tends to make this coincide with the ‘proper price,’ will it in the long run be profitable to him (New York Cotton Exchange et al., 1892: 50).

This statement’s imperative language – the speculator ‘must’ act in the way that is a ‘condition of his existence’ – underscores the subordination of the speculator to the market. Speculation required expertise, patience and a ‘well-equipped intelligence’ (US House of Representatives, 1910: 556). The only way a futures trader could profit was to keenly observe the spot market forces of supply and demand and, as his title would suggest, speculate on their likely impact on prices.

In the CBOT’s telling, this uni-directional relation between markets insulated spot prices from any effects of speculative buying and selling on the futures market. This view is clear in the Board’s position on the effects of speculative short sales, that is, instances where traders sold futures contracts, betting that prices would decline: ‘The short sale, when made, is not a transaction in cash grain … and since cash grain prices are determined by the present actual supply, the cash grain cannot be materially influenced by an offer to sell short for future delivery’.^11^ That is, since short sales do not alter spot market supply or demand, they cannot affect spot market prices. The same belief is evident in CBOT President Charles Hamill’s exchange with Representative William Hatch, the co-author of an 1892 anti-futures bill and chairman of the Congressional committee investigating its potential impact:

The CHAIRMAN: We will assume that for every actual bushel of wheat raised in the United States during 1892, there is, between the 1^st^ day of March and the 1^st^ day of September, 1,000 bushels of just such a proposition sold [in futures contracts] on the board of trade; do you think that has no effect in depressing the price of actual [spot] wheat?

Mr. HAMILL: Certainly it has none, in my judgment.

The CHAIRMAN: Then, according to your theory, the indefinite selling [of contracts] has no effect upon the actual product?

Mr. HAMILL: It does not. (US House of Representatives, 1892: 168)

Hammill argues that even ‘indefinite’ sales in the futures market would not affect the price of actual, physical wheat – speculators’ beliefs about what *might be* in the future could not change what *was* in the present. The only way that speculation could impact spot prices, according to CBOT representatives, was when short sellers who had been unable to set off their trades in the futures market were forced into the spot market to meet their contractual obligations.^12^ At these moments, the speculator, compelled by the impending due date of his futures contract, would ‘forcibly [become] a cash buyer’, adding to demand and thus raising prices in the spot market.^13^

The belief that speculation in futures markets emerged from and responded to spot market dynamics was further expressed in the CBOT’s belief that speculation integrated a homogenous, global market of profit-seeking actors. Speculators’ desire to gather more data and seek out speculative opportunities led to the expansion of market connections around the globe. Futures speculation ‘brought into existence the great grain markets of the world’ (US House of Representatives, 1892: 156–157) and ‘broaden[ed] the market’ (Sager, 1908: 12). The knowledge produced through this system of global speculation was unparalleled and ensured positive outcomes. As CBOT directors explained: ‘In an open world’s market, such as the grain market, downward manipulation is impossible. … Should the short seller offer grain below its legitimate value, the world’s buyers would flock to the market with their orders to take his offerings off his hands’.^14^ This claim succinctly brings together the multiple elements of the CBOT’s perspective on speculation: futures contracts promise real goods with a ‘legitimate value’ set by supply and demand, and enforced by the actions of calculative, profit-seeking speculators throughout the world.

But on the NOCE, where setting off was rather conceived as closing a parallel ‘insurance’ policy used to hedge spot market risk, ideas about speculation developed in a different direction. The ambiguous relation between spot and futures markets posited in the insurance view proved compatible with a perspective that saw bi-directional price relations between spot and futures markets, balkanized global markets and a variety of speculative motives.

NOCE members claimed that speculators’ motives were not only calculative, but also affective. They argued, for example, that Southern futures traders were ‘naturally in sympathy with Southern producers’,^15^ both for reasons of cultural affinity and because Southerners ‘produce the raw material and want to see it go higher’ (US House of Representatives, 1892: 107). A Southerner, unlike a trader on a foreign exchange, acted with ‘national pride or the national feeling towards the [cotton] producer’ (US House of Representatives, 1910: 635). Speculative prices existed amid a complicated mix of spirit, fear and anxiety, in addition to rational calculation. Spot traders treated futures as a barometer, so that a ‘demoralized’ futures market would make a spot trader anxious to sell, while a speculative advance would ‘stiffen him in his demands’.^16^ Vice-President EJ Glenny noted that the NOCE’s futures market gave comfort to the spot market actor, and ‘buoyed up his spirits to such an extent that he did not have to go into the market and sacrifice his product for fear that when he wanted to sell the other man would not want to buy’ (US House of Representatives, 1910: 636). According to the NOCE, futures prices did indeed influence local spot prices via their psychological effect on traders.

As the interests and emotions that motivated speculators’ actions were shared largely along geographic lines, the NOCE saw the global market not as integrated, but balkanized. A 1908 NOCE pamphlet argued:

Because Liverpool is the great market patronized by consumers, the predominant influence there is bearish, more men being interested in a declining market than in an advancing market. Because New Orleans is the great market patronized by producers, cotton bankers and merchants and by exporters … the predominant influence here is naturally bullish, more men being interested in an advancing market than in a declining market (New Orleans Cotton Exchange, 1908: 24).

Given these factions, the NOCE’s futures market was seen as a necessary counter-balance to those in Liverpool and New York, both of which were ‘affiliated with the spinning interests’ and ‘almost always do believe that prices will be lower, and to that end direct their efforts’ (Thompson, 1908: 19). The NOCE recognized both the divergent desires that existed across distinct regions and the power that masses of like-minded traders could exert over price. If legislators were to destroy the NOCE, ‘the only future market that looks and works for higher prices’ (Thompson, 1908: 19), they would ‘throw the balance of power into the hands of New York, Liverpool, Havre and other markets foreign to the cotton belt, which, to a greater or less extent, will thus be enabled to dictate to the South and to the entire world’ (New Orleans Cotton Exchange, 1908: 6). In contrast to the CBOT’s claim that price is set solely by spot market supply and demand, the NOCE offers a shockingly candid acknowledgment of (at least) tacit collusion in the setting of futures price based on shared interest.

In this section I have presented the culmination of the endogenous process by which the CBOT and NOCE developed divergent sets of ideas on the topic of speculation. The CBOT’s belief that setting off represented a simplified, ‘theoretical’ version of what might otherwise occur in the spot market formed the core of a perspective which insisted that price relations were uni-directional and that speculators were entirely calculative actors, whose thirst for profit integrated the world’s markets into a homogenous whole. Across all elements of this constellation, aspects of the futures market were consistently traced back to spot market roots, a connection itself enabled by the market ontology produced at the infrastructural level. In contrast, NOCE members looking at speculation saw bi-directional price relations across balkanized global markets, where members of competing exchanges were motivated by affect and desire, in addition to rationality. This perspective was enabled by the NOCE situating setting off within a process of ‘insurance’ involving parallel market positions. Their view on setting off, itself, had roots in the Exchange’s delivery infrastructure which failed to draw predictable, one-to-one linkages between spots and futures. The findings as a whole trace how differences in the CBOT and NOCE’s economic perspectives on setting off and futures speculation grew endogenously from the seeds of distinct infrastructures and the market ontologies they produced.

## Discussion and conclusion: Infrastructure, ontology and meaning

My findings show that the CBOT and NOCE constituted their derivative markets through dissimilar infrastructural connections with agricultural markets. These infrastructures established distinct ontologies of graded commodities, receipts and contracts. On the CBOT, graded commodities had an extant, physical reality, which could be referenced indexically in receipts and single-grade contracts. By contrast, graded commodities on the NOCE were only a potentiality that could not be referenced in a receipt, and whose contingency made contracts into symbolic promises rather than deals for identifiable items. The fact that graded commodities, as configured on the CBOT, could theoretically make a seamless transition from spot to futures markets and back again, enabled the Board to articulate an understandings of settlement by setting off as theoretical exchange, which the NOCE’s ambiguous border between spot and futures markets simply did not permit. These ontologies also gave form to two divergent constellations of ideas about the nature of speculation more broadly. On the CBOT, the quasi-mechanical relation between grades and contracts formed the core of a perspective that saw speculative dynamics entirely as an extension of spot market supply and demand. This view was less tenable on the NOCE, where the uncertain, mediated border between markets underpinned a view in which futures markets had their own dynamics and influence. The article demonstrates that infrastructures not only afforded the development of higher-order practices on derivative markets but also rooted and shaped the ideas with which those practices were understood.

What do we learn from these findings? A skeptical reply might claim that I have simply described a realist response in economic theory to two different types of markets: the CBOT and NOCE developed different economic ideas regarding speculation and setting off because these processes worked differently on their two markets. From this view, my findings are quite unsurprising.

I see two problems with this argument. First, it is empirically unsatisfying. As discussed above, the actual process of settlement by setting off was practically identical on the two markets – traders simply took on offsetting futures contracts and paid the difference between the two prices. So, to the extent that we consider setting off as a distinct process, arguing that it differed across the two markets is incorrect. More significantly, the exchanges’ theories cannot be reduced to realistic descriptions of differences in delivery infrastructures for the simple reason that they go beyond those differences. In the case of the NOCE, for example, the work of the Quotation Committee did require some local involvement in setting prices for futures deliveries; but this fact did not entail a broader belief in interest-based collusion on local markets. Or, on the CBOT, the physical presence of extant graded wheat made the notion of ‘theoretical delivery’ plausible but did not entail a uni-directional theory of price that strictly segregated present and future time frames. Rather, these theoretical positions emerged in creative processes of elaboration that were limited, but not determined, by infrastructural differences. The skeptical response conflates endogeneity with realism and exogeneity with constructivism, but my account is both endogenous *and* constructivist.

The second problem with this critique is that it presumes I am trying to determine *why* these exchanges developed distinct economic ideas. This is incorrect. Rather, what I want to ask is *how* market infrastructures shaped practitioners’ ideas about the nature of the market they faced. My answer to this ‘how’ question is that infrastructures produced market ontologies from which exchanges formed basic understandings of setting off and larger constellations of compatible ideas. To answer the ‘why’ question implicit in the skeptical response would require a broader study that closely traced the interaction of market infrastructures as endogenous ‘limiting causes’ (Wright et al., 1992) with the exogenous political, legal and cultural influences noted in prior scholarship (Cowing, 1965; Cronon, 1992; Fabian, 1999; Lurie, 1979; Stäheli, 2013).

With this skeptical response addressed, I turn to my positive contribution: illumination of the endogenous connection between infrastructures and ideas in derivative markets. As shown above, the key to this connection is considering infrastructures as productive of ontologies, which delimit the creative avenues available to practitioners in the development of economic theory. Extant scholarship focuses only on the surface level of ready-to-hand market devices, analyzing practitioners’ ideas as phenomenological outgrowths of mediated interactions (Aspers, 2007; Knorr Cetina, 2003; Preda, 2006; Zaloom, 2004) or pragmatic tools for making profit-focused interventions (Leins, 2018; MacKenzie, 2003; MacKenzie and Spears 2014; Svetlova, 2018). By contrast, my argument conceptualizes infrastructures as ‘emergent systems that produce novel configurations of the world – new practical ontologies … which give form to culture, society and politics’ (Jensen and Morita, 2017: 617-618). This ontological argument offers a way to analyse infrastructures’ impacts on knowledge production, despite their everyday invisibility to traders. The delivery infrastructures underlying the CBOT and NOCE’s futures markets were rarely utilized and uninvolved in speculative practices, yet the ontologies they produced shaped the development of economic ideas decades into the future. In proposing ontologies as ‘limiting causes’ (Wright et al., 1992) of practitioners’ economic ideas, the article opens a new avenue for investigating the endogenous impact of infrastructure on the production of knowledge in markets.

Beyond this direct theoretical contribution, these findings enable critical insights into two concepts used to study the relation of markets and ideas: *agencement* and performativity. First, the findings extend the ways in which ontology has been utilized in the literature on market *agencements* – mutually attuned, agentic assemblages of humans, discourses, procedures and technical devices (Callon, 2007). Work in this tradition, reflecting long-standing concerns in actor-network theory (Callon, 1986; Callon and Latour, 1992), most often uses ontology as a tool to analyse the nature of, and struggle over, agency. In this perspective, *agencements* ‘configure’ (Callon, 1998: 8) the ontologies of both objects and people, imbuing or depriving them of agency. Scholars have shown, for example, that commodities are stripped of agency when situated within *agencements* that transform them from unruly to ‘passive’ (Çalışkan and Callon, 2010) objects. Human agency is likewise determined by the range of tools and degrees of freedom available within an *agencement*, and can be configured at individual (‘the CEO’), collective (‘the firm’s employees’) or anonymous (‘market forces’) levels (Barry and Slater, 2005; Callon, 2008). The claims of this article go beyond this focus by showing that market ontologies additionally establish an object’s ideational boundaries, that is, how they can or cannot be incorporated into broader sets of economic ideas. This also reverses the usual nature of *agencement-*style analyses. These tend to approach ontology from the outside-in: the arrangement of the network as a whole configures the ontology of individual components. By contrast, my findings move from the inside-out: the ontology produced in a single infrastructure (e.g. for settlement by delivery) forms the foundation from which broader elements of an *agencement* (e.g. economic discourses) take shape. It thus suggests a more substantial analytical role for market ontologies in theories of markets as heterogeneous constructions.

This article also engages in a second reversal in relation to the core claims of the literature on performativity. Work on this topic has demonstrated how actors manipulate market devices (e.g. those involved in acts of measuring, calculating and representing) on the basis of academic theories, occasionally resulting in markets that hew more closely to the predictions of those very theories (Garcia-Parpet, 2007; Guala, 2001; MacKenzie, 2006). Economic ideas do not reflect a separate reality, but form the basis of interventions in reality (Hacking, 1983). My findings illuminate the reverse relation: Infrastructures, by establishing the core practices by which markets are performed, produce durable ways of thinking that can be elaborated outside of the immediate market setting. This illustrates a Durkheimian – as opposed to Austinian – style of performativity, whereby ritualized infrastructural practices (e.g. grading, receipting and contracting) objectify social relations into a market ontology that organizes and sustains collective beliefs (Appadurai, 2015; Durkheim, 2008; LiPuma, 2017; LiPuma and Lee, 2004). From this perspective, performativity serves not to embed ideas into the everyday materials of economic life, but to reify the materially-mediated practices of the market into economic conceptual frameworks. This Durkheimian style of performativity forms a meaningful complement to the literature’s main line of analysis.

Finally, beyond these critiques of *agencement* and performativity, the article also raises a significant question for the sociology of economic knowledge: How does market ontology as an endogenous ‘limiting cause’ interact with the myriad, exogenous, ‘forcing causes’ (Hirschman and Reed, 2014) impinging on the process of knowledge creation? As noted above, a hybrid style of analysis capable of addressing this question is a necessary prerequisite to answering the question of why any set of ideas emerges in a particular case. While this article has advanced an argument about infrastructures and ontologies as limiting causes, the larger goal remains to synthesize such arguments with sociology of knowledge-style approaches that recognize the impact of contextual factors. How flexible are market ontologies in terms of the ideas they can support? To what extent do ontologies to lose their limiting capacity over time or in the face of sustained external pressure? How do practitioners balance, incorporate or translate economic ideas whose ontologies conflict with the infrastructural features of their own markets? Efforts to answer these questions are likely to illuminate presently unrecognized tensions and relations between market infrastructures and the broader environment considered as elements in a sociology of economic knowledge.

## Notes

1. This style of analysis has been applied to economic ideas in multiple social settings and highlighted myriad sources of pressure, including politics (Backhouse, 2010; Bernstein, 2004; Mirowski, 1991, 2002), academia (Fourcade and Khurana, 2013; MacKenzie, 2006), broader institutional constellations (Erikson and Hamilton, 2018; Fourcade, 2009) and the culture at large (Dekker, 2016; Horn et al., 2011).

2. The one significant exception was the 1910 Scott Bill, which, shortly after its introduction, was amended to apply only to cotton and not grain. As will be seen, however, this extra pressure did not push the NOCE to adopt a more conciliatory position than the CBOT. So, if anything, this imbalance in the regulatory threat only strengthens the argument for endogenous influences.

3. The bulk of the CBOT’s legal proceedings in the last decades of the 19th century focused not on the legitimacy of speculation directly, but on the Board’s property rights over its price quotations (Lurie, 1979). Additionally, the timing of these court cases makes it hard to claim that they directly impacted economic ideas. The most famous case connected to the CBOT’s defense of speculation, *Christie v. Chicago Board of Trade*, began its life in the courts in 1900, decades after the genesis of futures trading and eight years after the Hatch and Washburn Bills were debated in Congress. While this case might have solidified certain key features of the CBOT’s theoretical perspective, the ideas preceded the legal confrontations. Generally speaking, it is not possible to pinpoint a unique element of the CBOT’s economic perspective that would have emerged in relation to the requirements of their legal battles.

4. Technically, the term for judging the overall quality of cotton is ‘classing’, not ‘grading’. In fact, I run the risk of confusing readers familiar with cotton markets, where the term ‘grade’ is used to refer only to a single facet of cotton’s quality, rather than its overall quality. However, I have chosen to use the term ‘grading’, despite its lack of technical precision, for the ease of non-specialist readers comparing across cases.

5. NOCE Annual Report, 1881. New Orleans Cotton Exchange records, Manuscripts Collection 652, Louisiana Research Collection, Howard-Tilton Memorial Library, Tulane University, New Orleans.

6. The one difference in the setting off process across these exchanges had to do with the number of different contracts available on the market. As noted above, the NOCE offered only a single standardized contract, while the CBOT offered a number of different contracts distinguished by grade. As a result, setting off on the CBOT required a contract-holder to find a partner willing to trade the *particular* graded contract he held, which setting off on the NOCE did not. In practice, this made little difference, as trading volumes were high enough on the CBOT that one generally could find a partner willing to trade for any given type of contract. The exception to this general pattern was when the market had been cornered, at which point demand would disappear (Cronon, 1992).

7. While the CBOT also praised the insurance function of hedging, it simultaneously insisted that eventually, somewhere, contracts ended in delivery of real goods – hence the language of ‘theoretical delivery’ and ‘buying back’ wheat seen in the previous section. Thompson and the NOCE made no such caveats, maintaining a distinction between spot deliveries and the legitimate non-delivery on hedging contracts.

8. Thompson 1910: 8. *Analysis and Exposition of the Scott Anti-Cotton-Future Bill: Depressing the Price of Cotton by Legislation.* New Orleans Cotton Exchange records, Manuscripts Collection, New Orleans Public Library, 219 Loyola Avenue, New Orleans.

9. Thompson, 1910 (see note 8): 8, emphasis added.

10. CBOT Annual Report, 1892. Chicago Board of Trade collection, Special Collections & University Archives, University of Illinois at Chicago Library, Chicago.

11. CBOT Board of Directors, 1921: 7. Untitled pamphlet on speculation. Chicago Board of Trade Collection, University of Illinois-Chicago.

12. CBOT Annual Report, 1893. Chicago Board of Trade Collection, University of Illinois-Chicago.

13. CBOT Board of Directors, 1921 (see note 11): 8.

14. CBOT Board of Directors, 1921 (see note 11): 7.

15. NOCE Annual Report, 1908: 12. New Orleans Cotton Exchange records, Tulane University.

16. Thompson, 1910 (see note 8): 12.
